# Genome assembly and annotation of the naked mole rat *Heterocephalus glaber* reared in Japan

**DOI:** 10.1038/s41597-026-06996-9

**Published:** 2026-03-18

**Authors:** Kouhei Toga, Kaori Oka, Hiroyuki Tanaka, Takehiko Itoh, Atsushi Toyoda, Hidemasa Bono, Kyoko Miura

**Affiliations:** 1 Laboratory of BioDX, PtBio Co-Creation Research Center, Genome Editing Innovation Center, Hiroshima University, 3-10-23 Kagamiyama, Higashi-Hiroshima city, Hiroshima 739-0046 Japan; 2 Laboratory of Genome Informatics, Graduate School of Integrated Sciences for Life, Hiroshima University, 3-10-23 Kagamiyama, Higashi-Hiroshima city, Hiroshima 739-0046 Japan; 3 Department of Aging and Longevity Research, Faculty of Life Sciences, Kumamoto University, 2-2-1 Honjo, Chuo-ku, Kumamoto city, Kumamoto 860-0811 Japan; 4 Department of Stem Cell Biology and Medicine, Graduate School of Medical Sciences, Kyushu University, Fukuoka, 812-8582 Japan; 5 Department of Life Science and Technology, Institute of Science Tokyo, Tokyo, 152-8550 Japan; 6 Comparative Genomics Laboratory, National Institute of Genetics, Yata 1111, Mishima, Shizuoka 411-8540 Japan; 7 Advanced Genomics Center, National Institute of Genetics, Yata 1111, Mishima, Shizuoka 411-8540 Japan

**Keywords:** Comparative genomics, Genome

## Abstract

The naked mole rat (NMR, *Heterocephalus glaber*) is a eusocial rodent that is native to northeastern Africa. NMRs exhibit extraordinary traits such as longevity, resistance to age-related decline, and remarkable hypoxia tolerance. Although the reference genome of this species has been determined because of its unique characteristics, the significance or role of intraspecific genomic variations remains unknown. In this study, we used PacBio long-read sequencing to generate a genome assembly of NMR reared in Japan. The assembled genome is 2.56 Gb. Benchmarking Universal Single–Copy Orthologs (BUSCO) revealed high completeness (95.2%). BRAKER3 estimated 26,714 protein-coding genes, and we successfully added functional annotations for 26,232 protein-coding genes using the functional annotation workflow. We identified 417 gene models that were previously undetectable in the reference genome of this species. We also identified structural and amino acid sequence variations between our assembly and the reference genome, suggesting the presence of intraspecific genomic variations. This new genomic resource could help uncover the molecular mechanisms underlying the behavioral and physiological traits of NMR.

## Background & Summary

The naked mole rat (NMR) has received significant attention in the field of biology because of its unique behavior and physiology. NMR is the longest-lived rodent, exhibiting extraordinary longevity and resistance to age-related diseases, including cancer, with a maximum lifespan of 40 years despite its small size^1^. This remarkably long lifespan deviates from the typical positive correlation observed between body mass and lifespan in mammals^2^. The NMR is a eusocial rodent that lives cooperatively in subterranean colonies consisting of a single breeding female, one to three breeding males, and non-reproductive workers^3^. Adapted to group-living in semi-enclosed subterranean environments, they exhibit significant tolerance to both hypoxia and anoxia^4^. Comparative genomic analysis between NMR and other mammals revealed that genomic mutations in NMR contribute to longevity, adaptation to the subterranean environment, and resistance to carcinogenesis^5–10^. Additionally, amino acid substitutions are enriched at disease-causing sites in human^6^. Therefore, the NMR genome is an important resource for identifying genetic factors related to its unique ecology, physiology, and resistance to age-related diseases.

The concept of a reference genome is based on a single representative genome sequence for each species and is broadly applied to many organisms, as seen in the Earth BioGenome Project^11^. However, this concept does not account for intraspecific variation. Therefore, it remains unclear how genomic structures are preserved within the same species. Supporting these limitations, intraspecific genomic variations and chromosomal rearrangements have been observed in mammals, larvaceans, and insects^12–14^. Notably, maximal genetic distances within NMR species are approaching interspecific values observed among other Bathyergid species, based on calculations for the cytochrome-b gene between the Ethiopian and southern Kenyan populations^15^. This suggests the accumulation of genetic differences within the NMR species. Additionally, the rivers in Kenya may cause reproductive isolation, leading to local genetic differences^16^. However, genome resources are currently insufficient to identify genomic differences in NMR species. To address this gap, we sequenced the whole genome of a single NMR individual reared in Japan using Illumina short reads and PacBio long reads. Gene prediction and functional annotation were performed using BRAKER3^17^ and the functional annotation workflow (Fanflow)^18^ pipeline. We verified the presence of variations at both the genome and gene levels between NMR species, demonstrating that our genome assembly provides a valuable resource for advancing the understanding of genomic functions related to NMR physiology and behavior.

## Methods

### Sample collection, sequencing, and genome assembly

**Fig. 1:**
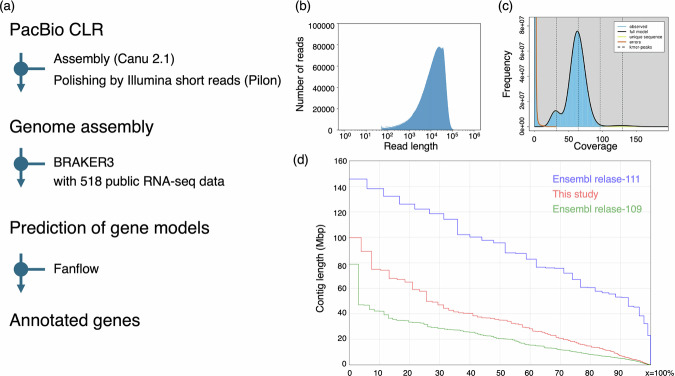
Strategies for genome assembly and annotation in this study (**a**). The main tools adopted in assembly and annotation are described. Histogram of read lengths after log transformation (**b**). GenomeScope2 results for genome size, heterozygosity rate, and repeat sequences (**c**). Heterozygous and homozygous peaks are shown by the first and second peaks, respectively. Nx plots for our assembly and Ensembl assemblies (release-111 and −109) (**d**). Different colored lines indicate different assemblies.

NMRs were maintained at 30 ± 0.5 °C and 55 ± 5% humidity with 12 h light and 12 h dark cycles at Kumamoto University. All animal experiments were performed in accordance with the Guide for the Care and Use of Laboratory Animals (United States National Institutes of Health). The ethics committee of Kumamoto University approved all the procedures (approval No. A2020-042, A2022-079 and A2024-063). The naked mole-rats (Heterocephalus glaber) used in this study originate from colonies previously maintained in the laboratory of Dr. Kazuo Okanoya (formerly at RIKEN, now at Teikyo University). These animals are derived from two lineages that were exported from northeastern Africa before 1992 and subsequently bred and maintained in research institutions and zoos in South Africa and the United States. Although the exact geographic origin of the wild-caught founders is unknown, they are believed to have come from the species’ native range, which spans Ethiopia, Kenya, Somalia, and Djibouti. Since 2011, the colony has been continuously maintained in our laboratory. Genomic DNA was extracted from the quadriceps muscle tissues from a male NMR using a QIAGEN Genomic-tip (QIAGEN, Hilden, Germany) according to the manufacturer’s instructions. Our strategy of genome assembly and the annotation was described (Fig. 1a). The quality and quantity of the extracted genomic DNA were assessed using a Qubit 4 fluorometer (Thermo Fisher Scientific, Waltham, MA, USA) and a Pippin Pulse system (Agilent Technologies). DNA was sheared into 30–100 kb fragments using a g-tube device (Covaris Inc., MA, USA). A continuous long-read (CLR) single-molecule real-time (SMRT) bell library was prepared using the SMRTbell Express Template Prep Kit 2.0 (Pacific Bioscience, Menlo, CA, USA) according to the manufacturer’s instructions. The CLR libraries were size-selected using the BluePippin system (Saga Science, MA, USA) with a lower cutoff of 30 kb. The library was run on the PacBio Sequel II platform using two SMRT Cell 8Ms with Binding Kit 2.0, and Sequencing Kit 2.0 with 20-h movies. A total of 15.2 M subreads were generated from two SMRT cells, yielding 282.41 Gb raw data (109.9-fold whole-genome coverage). The average read length of CLR was 18.5 kb (Fig. 1b). Additionally, genomic DNA was fragmented to an average size of 500–600 bp using a focused ultrasonicator M220 (Covaris Inc., MA., USA). Paired-end libraries with 450–550 bp insert sizes were constructed using the TruSeq DNA PCR-Free Library Prep Kit (Illumina, CA, USA) and size-selected on an agarose gel using a Zymoclean Large Fragment DNA Recovery Kit (Zymo Research, CA., USA). The final libraries were sequenced following the 2 × 250 bp paired-end protocol for NovaSeq. 6000 systems (Illumina, San Diego, CA, USA), yielding 429.39 Gb of raw data. This data size corresponds to 167.13 times the genome coverage. GenomeScope2^19^ (*k* = 32) estimated the genome size to be 2.569 Gb, with 18% consisting of repeat sequences (Fig. 1c). The rate of heterozygosity was 0.173%.

Canu 2.1^20^ was used for *de novo* assembly of PacBio CLR reads with parameters ‘-genomesize 3.0 g -coverage 200 -minlength 1000 -raw -pacbio’. Three rounds of polishing were performed using Pilon^21^ with default settings and with the aforementioned Illumina short reads. Purge_dups^13^ was used with default parameters on the polished assembly to purge haplotigs, resulting in a genome size of 2.561 Gb, which was comparable to that estimated by GenomeScope2^19^ (Table 1). Merqury v1.3 (*k* = 21)^22^ was used for the validation of our genome assembly. Completeness and quality value was 98.2% and 50.6, respectively. We compared the assembly statistics of the current and conventional assemblies in Ensembl release-111^23^ and release-109^24^ (Table 1 and Fig. 1d). Contiguity of our genome assembly was inferior to that of the current Ensembl genome (release-111), but Benchmarking Universal Single–Copy Orthologs (BUSCO)^25^ showed our assembly had the highest completeness (95.2%).

**Table 1:** Comparison of assembly statistics with reference genomes in Ensembl.

	parameter	this study	Ensembl release-111	Ensembl release-109 (HetGla_female_1.0)
Assembly statistics	Length	2,561,275,506	2,500,150,894	2,618,204,639
	N50	35,055,060	95,752,777	20,532,749
	L50	25	11	42
	No. of contigs or scaffold	383	32	4,229
	Largest contig	99,770,558	145,805,425	78,885,850
	GC %	40.01	39.97	35.55
BUSCO results (OrthoDB v10 Glires database)	Complete [single, duplicated]	95.2% [90.6%,4.6%]	92.6% [88.2%,4.4%]	94.7% [90.3%,4.4%]
	Fragmented	0.8%	1.2%	1.0%
	Missing	4.0%	6.2%	4.3%

### Gene prediction and functional annotation

Since repetitive sequences can lead to overestimation of gene predictions, repetitive sequences were filtered using RepeatModeler v2.0.3 and RepeatMasker v4.0.6 in Extensive *de novo* TE Annotator v2.0.1^26^, and were soft-masked. We identified 978 Mb of repetitive sequences, accounting for 38.19% of the genome assembly^27^. Gene prediction was performed using BRAKER3^17^. Public RNA sequencing (RNA-Seq) data^28^ and Vertebrate protein dataset (https://bioinf.uni-greifswald.de/bioinf/partitioned_odb11/, accessed on October 10, 2023) were used as extrinsic evidence for BRAKER3^17^. Metadata of public RNA-Seq data was collected using SRA Run Selector in National Center for Biotechnology Information (NCBI), and manually filtered to retain only samples with well-defined metadata. Sequence read archive (SRA) retrieval of public RNA-Seq and conversion to FASTQ files was performed using SRA toolkit v3.0.5 (https://github.com/ncbi/sra-tools, accessed on June 30, 2023). Trimming and quality control of the FASTQ files were performed using Trim Galore! v.0.6.10^29^ with ‘–fastqc,–trim1’ option. Mapping RNA-Seq reads to the genome was performed using HISAT2 v2.2.1^30^ with ‘-q,–dta’ option, and then obtained SAM files were converted to BAM files using SAMtools v1.12^31^. The obtained BAM files were supplied to the BRAKER3 workflow. Gene set coverage was assessed using BUSCO v5.2.2^25^ with ‘-l glires_odb10.’ BRAKER3 predicted 26,714 protein-coding genes in the assembly (Table 2). BUSCO completeness values were comparable to those of the current and conventional genomes in Ensembl.

**Table 2:** Comparison of gene prediction statistics between our assembly and the reference genome in Ensembl.

	Parameter	This study	Ensembl release-111	Ensembl release-109
	The number of protein-coding genes	26,714	20,742	23,216
	The number of mRNA	43,234	41,841	72,114
BUSCO results (OrthoDB v10 Glires database)	Complete [single, duplicated]	92.2% [49.2%,43.0%]	90.1% [38.6%,51.5%]	93.8% [68.6%,25.2%]
	Fragmented	0.8%	1.9%	1.2%
	Missing	7.0%	8.0%	5.0%

Fanflow^18^ was used as the reference protein set in Ensembl release-111. This workflow used GGSEARCH v36.3.8 g in the FASTA package (https://fasta.bioch.virginia.edu/, accessed on July 19, 2023) to compare amino acid sequences against datasets from humans, mice, guinea pigs, female NMR, male NMR, and UniProtKB/Swiss-Prot. Domain searches were conducted using HMMSCAN in HMMER v3.3.2, with Pfam v35.0, which served as the domain database^32^. Of the 26,714 predicted genes, 98% (26,232/26,714) showed matches to at least one protein in the reference protein sets or the Pfam database, leaving only a small number of unclassified proteins (Table 3).

**Table 3:** Summary of the functional annotation results.

Annotation category	Reference	The number of gene annotated
1. Gene homolog (GGSEARCH)	Human	18,730
	Mouse	18,251
	Guinea pig	17,080
	NMR (female)	17,897
	NMR (male)	17,983
	UniProtKB/Swiss-Prot	19,149
	At least one of the above	26,120
2. Genes annotated only by HMMSCAN	Pfam domain database	112
1 + 2	—	26,232
Hypothetical protein	No hit	482
Total		26,714

### Comparison of gene models between our assembly and the Ensembl assembly

During Fanflow execution, 417 transcripts with counterparts in the protein sets of all Ensembl reference species were found, except for those in NMR (female and male). This implied that these transcript models were not present in the Ensembl NMR genome. To confirm whether these 417 transcripts were missing from the NMR genome in Ensembl, we ran miniprot^33^ to map the translated proteins predicted from our assembly onto the female NMR genome in Ensembl. Subsequently, bedtools intersect (v 2.31.1)^34^ with ‘-s -loj’ options was used to identify overlapping regions between the mapped proteins and GFF3 file of the female NMR genome in Ensembl release-111 (accessed on May 30, 2024). Amino acid sequences were aligned using MAFFT^35^ with ‘–auto’ option, and then the alignments were visually examined to evaluate the differences of gene structures between our assembly and the female NMR genome in Ensembl.

**Fig. 2:**
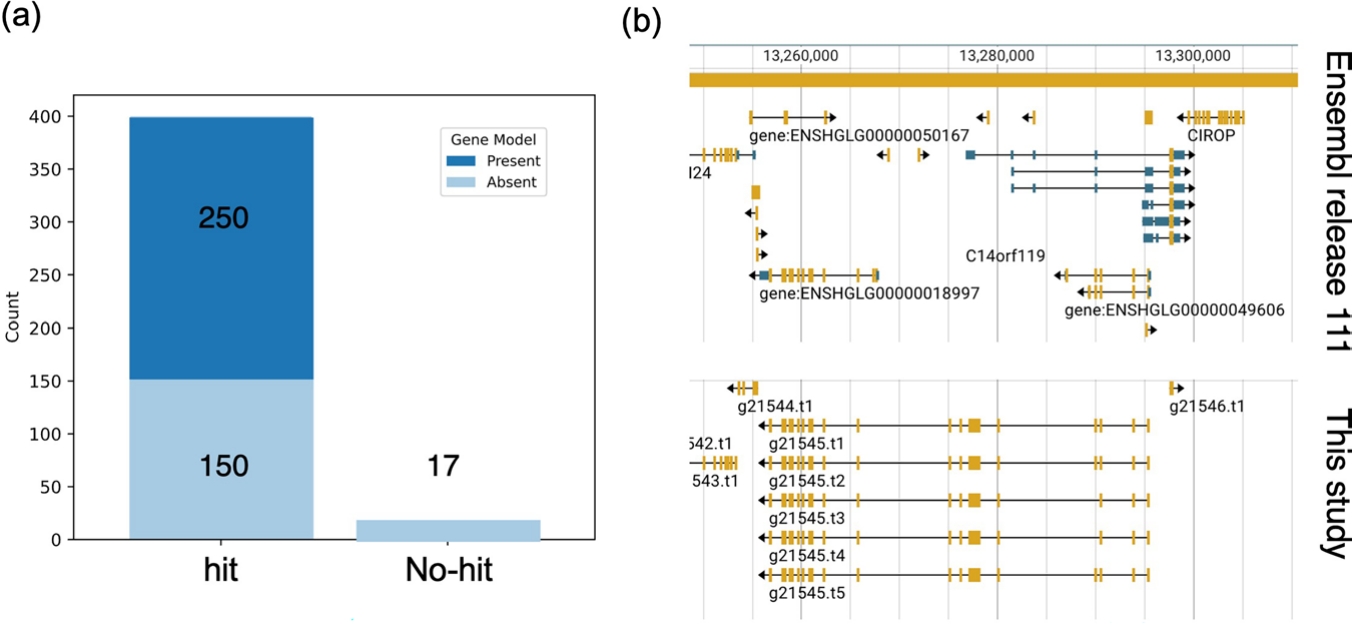
Searches for the gene model differences between our assembly and the Ensembl assembly (release-111). The number of regions mapped by miniprot is shown, with columns colored differently to indicate the presence of overlaps with the Ensembl gene model (**a**). Gene structures of g21545 and their corresponding regions in the Ensembl genome are shown (**b**). Yellow and blue boxes indicate the exons and untranslated regions (UTRs), respectively.

Of the 417 transcripts, 400 were mapped to the female NMR genome in Ensembl, suggesting that these 400 transcripts are encoded in Ensembl assembly. The 400 transcripts were further categorized into two groups: one consisting of those present in the gene model of the female NMR genome in Ensembl (250 transcripts), and the other consisting of those absent from the gene models (150 transcripts) (Fig. 2a)^36^. Among the 250 transcripts, 39 overlapped across multiple gene models in the female NMR genome of Ensembl (e.g., the g21545 gene, as shown in Fig. 2b). The exon of g21545 overlapped with those of ENSHGLG00000049606 and ENSHGLG00000018997. Meanwhile, remaining 17 transcripts were not mapped by miniprot (Fig. 2a)^36^, suggesting that that these transcripts might be absent from the Ensembl assembly. These results show that previously undetected gene models in the Ensembl genome were included in our assembly.

### Comparison of genomic differences between our assembly and the Ensembl assembly

**Fig. 3:**
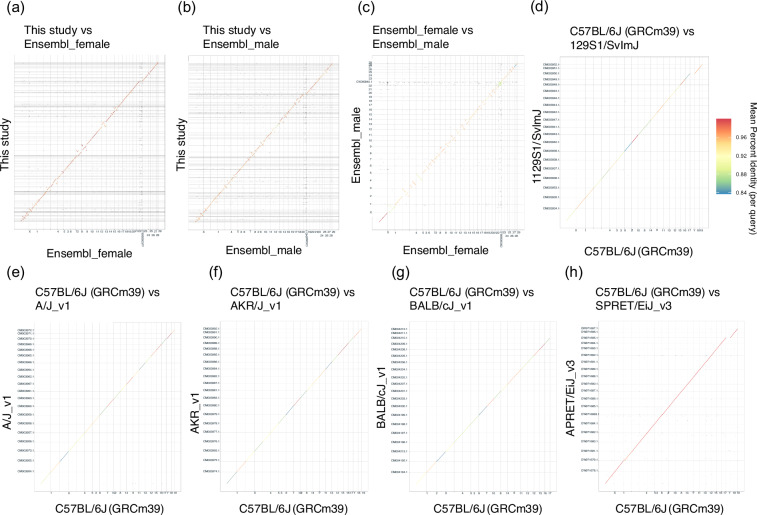
Differences in genome structures within or between species. Dot plots indicate the comparisons of genome structures between our assembly and Ensembl assembly release-111 (female and male) (**a**–**c**), between C57BL/6 J (GRCm39) and each mouse strain (**d**–**g**), and between C57BL/6 J (GRCm39) and SPRET/EiJ (*Mus spretus*) (**h**). The color of plots indicates the mean percent identity (per query).

Dot plots were generated to visualize the genomic differences between our assembly and the Ensembl assembly^23,37^. To evaluate genomic differences within the NMR, dot plots were generated among mouse strains (C57BL/6 J vs. 129S1/SvilmJ_v1, C57BL/6 J vs. A/J_v1, C57BL/6 J vs. AKR_J_v1, and C57BL/6 J vs. BALB_cJ_v1) and between mouse species (C57BL/6 J vs. *Mus spretus* (SPRET/EiJ)). The same process was applied to combinations of mouse strains to evaluate intraspecific differences in NMR. C57BL/6 J in Ensembl release-111^38^, GCA_001624185.1 (129S1/SvImJ_v1)^39^, GCA_001624215.1 (A/J_v1)^40^, GCA_001624295.1 (AKR/J_v1)^41^, GCA_001632525.1 (BALB/cJ_v1)^42^, and GCA_921997135.2 (SPRET_EiJ_v3)^43^ were used in this analysis. Minimap2 v2.28^44^ was used to align genome sequences with the ‘-cx asm5’ option. Subsequently, dot plots were visualized using dotPlotly with ‘-s -t -m 500 -q 500000 -l’ options (https://github.com/tpoorten/dotPlotly, accessed on July 17, 2023). Several inversions were observed in the NMR genome, but most plots showed a nearly linear pattern (Fig. 3a–c). In contrast, the dot plot between mouse strains and mouse species showed an almost completely linear pattern (Fig. 3d-g). The presence of gaps at chromosomes 18 and 19 in Ensembl was manually checked to confirm false positive inversions. All inversions examined occurred near gaps regions^45,46^. These results suggest that intraspecific chromosomal variations may occur due to scaffold assembly errors.

In addition, structural variants (SV) between our assembly and the Ensembl assembly were searched. Minimap2 v2.28^44^ with ‘asm5–cs -r2k’ option was used to map our assembly to female NMR assembly in Ensembl. Svim-asm v1.0.3^47^, using the default option, was used to identify SVs greater than 40 bp. As a result of Svim-asm, 2,263 deletions and 2,668 insertions were identified between our assembly and the Ensembl assembly (Table 4)^48^. To evaluate these statistics, we examined the SVs between the mouse strains (Table 4). The number of SVs (deletions or insertions) exceeded 100,000 in all mouse pairs examined in this study. The number of SVs (deletions or insertions) was lower in NMR than in the mouse strains, indicating that structural variations within NMR may be smaller than those within mouse strains. Intraspecific variations generated by Minigraph-Cactus^49^ were also available^50^. This program revealed 1,090,118 variations including SNPs and SVs between our assembly^51^ and Ensembl assembly^23^. In addition, the SVs were searched using PacBio raw reads by Sniffles^52^. Detected deletions and insertions were increased compared with assembly-based methods of SV search (Table 5). Obtained VCF files are available in European Variation Archive^53,54^.

**Table 4:** Structural variants within NMR and within mice.

SV	This study vs Ensembl	C57BL/6 J vs 129S1/SvImJ_v1	C57BL/6 J vs A/J_v1	C57BL/6 J vs AKR/J_v1	C57BL/6 J vs BALB/cJ_v1
Inversion	40	50	64	70	64
Deletion	2,263	104,041	113,225	119,318	112,688
Insertion	2,668	18,614	17,804	18,583	16,961
Tandem duplication	0	0	0	0	0
Interspersed duplication	0	0	0	0	0
Breakend	0	0	0	0	0

**Table 5:** Structural variants within NMR for SV search using raw reads.

SV	Our assembly vs our raw reads	Ensembl assembly vs our raw reads
Inversion	47	219
Deletion	2023	5323
Insertion	41,013	42263
Duplication	27	95
Breakend	589	1019

### Search for sequence differences of genes between our assembly and the Ensembl assembly

To compare the gene sequences between our genome assembly and the female NMR assembly in Ensembl, the gene annotations of our assembly were transferred to the female NMR assembly in Ensembl using Liftoff v1.5.2^55^. Transferred gene annotations were filtered using the parameter “valid_ORF = True” and “match_ref_proteins = FALSE” to identify transcripts with intraspecific variations of translated amino acid sequence between our assembly and female NMR assembly in Ensembl. The same processes were applied to combinations of mouse strains (C57BL/6 J vs. 129S1/SvilmJ_v1, C57BL/6 J vs. A/J_v1, C57BL/6 J vs. AKR_J_v1, and C57BL/6 J vs. BALB_cJ_v1) to evaluate intraspecific differences in NMR. C57BL/6 J in Ensembl release-111^38^, GCA_001624185.1 (129S1/SvImJ_v1)^39^, GCA_001624215.1 (A/J_v1)^40^, GCA_001624295.1 (AKR/J_v1)^41^, and GCA_001632525.1 (BALB/cJ_v1)^42^ were used in this analysis. The transcripts with intraspecific variations in NMR were filtered using the Liftoff parameter “Sequence_ID,” which indicates the sequence identity. The transcript ID of NMR and each mouse strain were mapped to those of the C57BL/6 J protein ID using the annotation results from Fanflow^56^ and Ensembl Biomart, respectively. Intervene^57^ (accessed October 18, 2024) was used to identify the intersections of the gene sets. Enrichment analysis was performed using the Metascape software with default settings (https://metascape.org/, accessed on October 25, 2024)^58^.

**Fig. 4:**
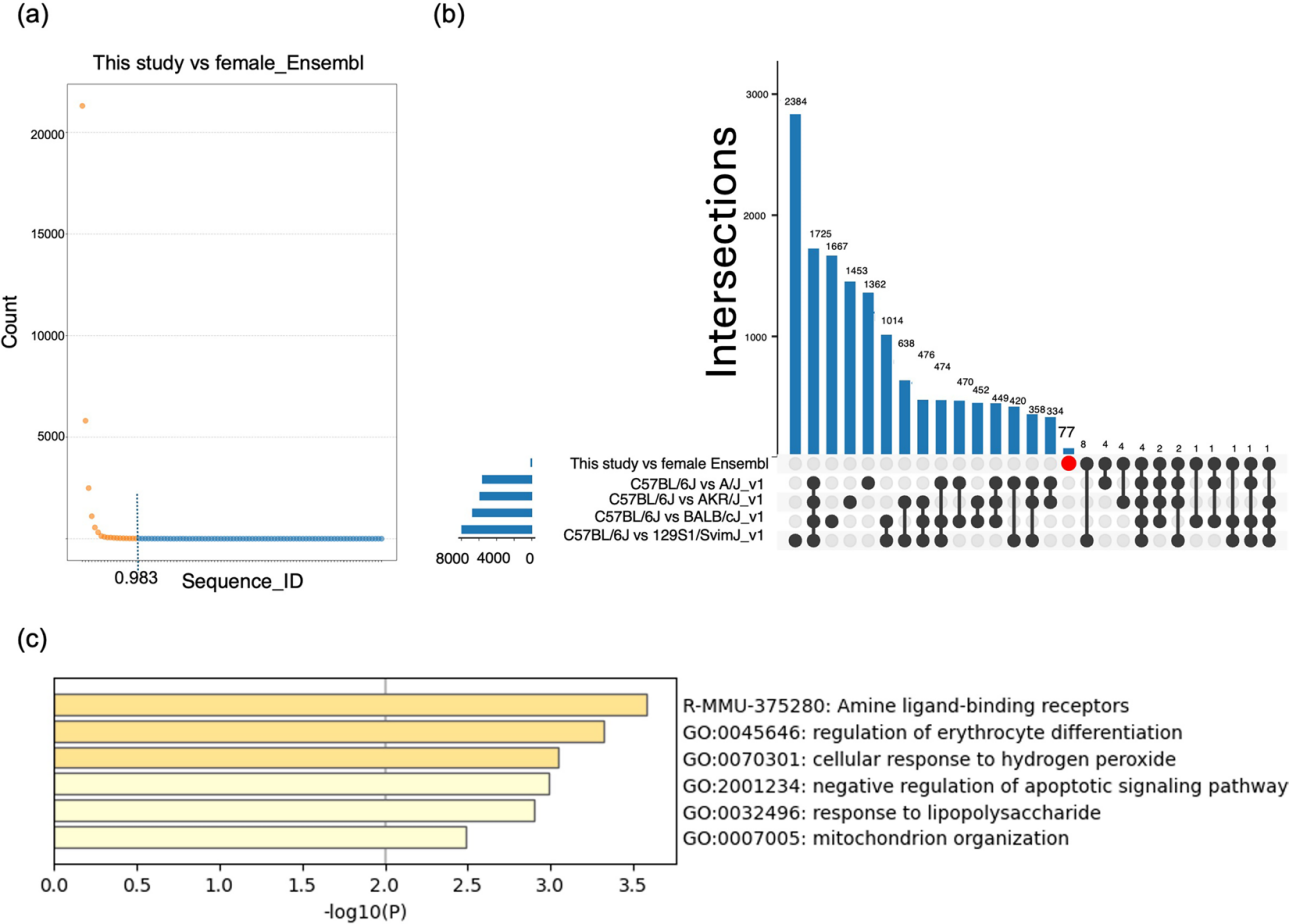
Mutations for amino acid sequences between our assembly and the female Ensembl assembly. (**a**) Total numbers of transcripts per Sequence_ID. The dot color changes based on a Sequence_ID threshold of 0.983. (**b**) Intersections of transcripts with intraspecific variations in NMR and mouse strains. Red circle indicates transcript sets with intraspecific differences only within NMR. (**c**) Enriched terms in NMR-intraspecific 77 transcripts indicated by red circle in (**b**).

The annotation of 32,263 transcripts from our assembly was successfully transferred to the female NMR assembly in Ensembl, and sorted by indicators of sequence identity, “Sequence_ID” (Fig. 4a). In the NMR comparison, 177 transcripts had a “Sequence_ID” below 0.983, and the count per “Sequence_ID” below this threshold was consistently under 10, suggesting that intraspecific variation in these transcripts was pronounced in the gene sets analyzed in this study. When comparing intraspecific transcripts between NMR and mouse strains, 77 genes exhibited NMR-specific intraspecific variations (Fig. 4b)^59^. Enrichment analysis of the 77 transcripts revealed significant enrichment of genes involved in amine ligand-binding receptors, including 5-hydroxytryptamine (serotonin) receptor 2B (*Htr2b*), G protein-coupled receptor 143 (*Gpr143*, a receptor for L-3,4-dihydroxyphenylalanine [L-DOPA]), erythropoietin (*Epo*), and thyroid hormone receptor beta (*Thrb*) (Fig. 4c)^60^. Intraspecific variations in *Htr2b* and *Gpr143* suggest that responses to serotonin and L-DOPA may differ even within NMR populations, potentially influencing social behavior. Notably, NMR fibroblasts exhibit unique serotonin management, in which serotonin accumulates and induces cell death through serotonin metabolism in response to cellular senescence^61^. Although SVs were also detected in these gene loci, they were located in intron regions^62^. Additionally, intraspecific variations in *Epo* and *Thrb* may contribute to adaptation to hypoxic underground environments through changes in erythropoiesis and metabolic regulation. Examining the functions of these genes could help deepen our understanding of the molecular mechanisms underlying the unique traits of NMRs, such as eusociality, hypoxia tolerance, and delayed aging. Overall, genes exhibiting intraspecific variations may provide valuable insights into the behavioral and physiological adaptations of NMR.

## Data Records

All raw sequence data for genome assembly (Illumina short reads and PacBio CLR) have been deposited in the DNA Data Bank of Japan under accession numbers DRR401650^63^, DRR401651^63^, DRR401652^63^ and DRR401653^63^. Genome assembly and WGS projects were deposited under the accession numbers GCA_053883595.1^51^ and BAAHMU010000000-BAAHMU010000382, respectively. Full information on functional annotations added using Fanflow is available at *figshare*^56^. Structural variants within NMRs have been deposited in EVA^48,50,53,54^. Gene models estimated by BRAKER3 for our assembly have been deposited in *figshare*^64^.

## Technical Validation

The performance of the assemblers was evaluated by comparing four assemblers. The four assemblers (wtdbg2^65^, Flye v2.8^66^, Canu v2.0^20^, and Canu v2.1^20^) were used to compare the assembly quality with respect to contiguity. The assembly, constructed by Canu v2.1, had the longest average and maximum lengths in contigs, and showed the minimum L50 value (Table 5). Pilon was used to polish the genome assembly constructed using Canu v2.1, and Purge_dups^67^ was used to remove haplotigs. This process improved genome statistics, particularly regarding contiguity (Table 6).

**Table 6:** Comparison of genome assemblers.

Assembler	wtdbg2	Flye v2.8	Canu v2.0	Canu v2.1
Genome size (Gb)	2.519	2.555	2.741	2.683
No. of contigs or scaffold	4196	1450	1855	1459
Average length (Mb)	0.6	1.76	1.46	1.84
Max length (Mb)	73.97	71.69	93.24	99.77
N50 (Mb)	18.64	24.74	36.24	34.94
L50	43	34	27	26
Gap	0%	0.00%	0%	0%

## Data Availability

All raw sequencing data generated in this study, including Illumina short reads and PacBio CLR long reads, have been deposited in the DNA Data Bank of Japan (DDBJ) Sequence Read Archive under accession numbers DRR401650–DRR401653 (https://ddbj.nig.ac.jp/search/entry/sra-study/DRP012667)^63^. The genome assembly has been deposited under accession GCA_053883595.1, and the whole-genome shotgun (WGS) project under BAAHMU010000000–BAAHMU010000382. Functional annotation results produced using Fanflow (TSV format) and gene models predicted by BRAKER3 (GTF format and FASTA format) are available on figshare (10.6084/m9.figshare.28171166 and 10.6084/m9.figshare.28180430). The Fanflow annotation file includes the following columns: braker_id (BRAKER3 transcript ID), human_pid / human_gene / human_description (human best-hit protein, gene symbol, and description), mouse_pid / mouse_gene / mouse_description (mouse best-hit protein, gene symbol, and description), guinea_pig_pid / guinea_pig_gene / guinea_pig_description (guinea pig best-hit protein, gene symbol, and description), Hgla_female_pid / Hgla_female_gene / description (Naked mole-rat female best-hit protein, gene symbol, and description), Hgla_male_pid / Hgla_male_gene / description (Naked mole-rat male best-hit protein, gene symbol, and description), uniprot_id / uniprot_description (UniProtKB best-hit protein and description), pfam_id / pfam_description (Pfam domain IDs and domain annotations). Structural variants identified in this study (VCF format) have been deposited in the European Variation Archive (EVA) under project accession numbers ERZ28787178 (https://identifiers.org/ena.embl:ERZ28787178)^53^, ERZ28787179 (https://identifiers.org/ena.embl:ERZ28787179)^54^, ERZ28787181 (https://identifiers.org/ena.embl:ERZ28787181)^48^, and ERZ28787182 (https://identifiers.org/ena.embl:ERZ28787182)^50^. All datasets are publicly accessible at the repositories listed above.

## Code availability

All programs and pipelines were executed following their official manuals or help pages. Version and parameter information used in our analysis are provided in the Methods section. No custom scripts were used.
